# Multiscale Modeling and Analysis of Muscle Tissue: A Finite Element Approach for 3D Braided Composite Structures

**DOI:** 10.3390/biomimetics11060396

**Published:** 2026-06-04

**Authors:** Vivek Kumar Dhimole, Niraj Kumar, Seul-Yi Lee, Soo-Jin Park

**Affiliations:** 1 School of Computer Science and Artificial Intelligence, SR University, Warangal 506371, Telangana, India; vivek.dhimole@sru.edu.in; 2 Department of Mechanical Engineering, College of Engineering, Kyung Hee University, Yongin 17104, Republic of Korea; 3 Department of Convergent Biotechnology and Advanced Materials Science, Kyung Hee University, Yongin 17104, Republic of Korea

**Keywords:** biomechanics, computational mechanics, multiscale modeling, muscles, stretch

## Abstract

Skeletal muscle mechanics arise from hierarchical fiber–matrix interactions spanning multiple length scales. This study presents a computationally efficient, composite-inspired multiscale finite element framework that links muscle microstructure to whole-muscle behavior through explicit periodic numerical homogenization. Muscle fibers and endomysium are resolved at the microscale, their homogenized response is propagated to fascicles embedded in the perimysium at the mesoscale, and the resulting properties are incorporated into a three-dimensional macroscale muscle model including the epimysium. Unlike phenomenological continuum models or computationally intensive chemo-electro-mechanical approaches, the proposed framework enables scalable three-dimensional simulations while preserving microstructural load-transfer mechanisms. The predicted stress–strain relationships in uniaxial tensile loading were in agreement with experimental values, with differences of about 1–3%. Passive elasticity of muscle is simulated in the present research in order to provide the computation model as a benchmark in further development into the active contraction and the viscoelastic behavior. Additionally, it provides a modeling basis for patient-specific studies under varied pathological conditions.

## 1. Introduction

Skeletal muscle exhibits a highly organized hierarchical architecture in which force-generating muscle fibers are embedded within a connective tissue matrix composed of the endomysium, perimysium, and epimysium [1,2]. At the microscale, individual muscle fibers are surrounded by endomysium, and groups of fibers form fascicles encased by the perimysium [3]. At the macroscale, the entire muscle is enveloped by the epimysium [4]. Although connective tissue constituents occupy a relatively small fraction of muscle volume, they play a critical role in governing the passive mechanical behavior, force transmission between fibers, and overall structural integrity of muscle tissue [5,6]. Consequently, the macroscopic mechanical response of skeletal muscles emerges from complex interactions across multiple length scales.

Accurate characterization of skeletal muscle mechanics is essential for understanding normal function, injury mechanisms, and disease-related alterations, such as muscular dystrophy, spasticity, and fibrosis [7,8]. From a biomechanical perspective, muscle behavior is inherently nonlinear, anisotropic, and strongly influenced by microstructural organization and fiber orientation [9,10]. These features have motivated the development of continuum mechanics-based constitutive models in which skeletal muscles are often represented as fiber-reinforced composite materials [11,12,13,14]. These models have been successfully implemented within finite element (FE) frameworks to investigate muscle deformation, stress distribution, and architectural effects at the tissue and organ levels [15,16,17,18].

Despite these advances, many existing muscle models rely on phenomenological constitutive descriptions or simplified homogenization assumptions that do not explicitly resolve underlying microstructures. Classical estimates, such as Voigt-type homogenization, are computationally efficient and perform adequately under loading conditions aligned with the muscle fiber directions [19,20,21]. However, such approaches neglect the detailed spatial arrangement of fibers and the extracellular matrix (ECM), limiting their predictive capability under complex loading states involving shear, transverse deformation or large strain heterogeneity. As the mechanical contrast between the muscle fibers and connective tissue increases, these limitations become increasingly pronounced.

To overcome these shortcomings, several studies have introduced microstructure-informed computational methods. Finite element models based on histological cross-sections have been used to estimate effective shear properties [22,23], investigate deformation-induced muscle damage [24,25], and explore disease-related microstructural changes [26,27]. More recent studies have incorporated hierarchical muscle architecture to predict force–velocity behavior and fiber-type interactions [28,29]. Although these studies represent important progress, they are often limited to a single scale, restricted geometries, or computationally expensive frameworks that hinder extension to whole-muscle simulations.

Accordingly, there remains a need for computationally efficient multiscale modeling strategies that (i) explicitly account for the muscle microstructure, (ii) enable systematic homogenization from the fiber to whole-muscle scale, and (iii) are readily implementable within standard FE environments [30,31]. In particular, approaches that borrow concepts from the multiscale modeling of heterogeneous engineering materials, such as textile composites, offer promising yet underexplored pathways for skeletal muscle analysis. These methods provide structured strategies for representing fiber packing, matrix interactions, and hierarchical homogenization across various length scales.

The relationship between the structure of skeletal muscles and composite structures in engineering is not only a comparison but a demonstration of an underlying biomimetic approach: nature has evolved the same hierarchical fiber/matrix structure that engineers have created for advanced structural composites. The skeletal muscle consists of force-producing fibers imbedded in a flexible matrix at the microstructure level, which are then bundled into fascicles surrounded by the perimysium at the mesostructure level, and wrapped with the epimysium at the macrostructure level—a three-level hierarchical organization identical to that of braided textile composites [32,33,34]. Biomimetics can be defined broadly as a double-edged approach that includes design transfer from biology to engineering as well as engineering analysis applied to biological systems. Such an approach is adopted in the current investigation, wherein the rigorously developed multiscale periodic homogenization theory used traditionally in the modeling of heterogeneous composite materials is utilized to represent the hierarchical biological structure of skeletal muscle. Thus, it facilitates microstructure-based modeling in a way that is challenging to attain using purely biological models. The transfer of such an established technique from one field of research to another is, therefore, the main biomimetic approach adopted in this investigation.

In addition to providing insight into the mechanics of skeletal muscle tissue itself, there is also a future-oriented set of implications regarding biomimetic engineering in the field. Modern soft actuators, artificial muscles, and bio-hybrid robots are constantly aiming at mimicking the properties of biological muscle tissue, including such aspects as compliance, anisotropicity, and hierarchical load distribution [2,3]. The problem, however, is that such design of a system as choosing appropriate values of fiber volume fraction, matrix stiffness, fascicle orientation, and the architecture of connective tissue requires the kind of quantitative relations between microstructure and mechanical properties of the material that the current multiscale homogenization approach can provide. The ability to predict how the alteration of the endomysium, perimysium, and epimysium will affect the macroscopic mechanical properties of a muscle opens up opportunities in the design of biomimetic composite materials. In other words, this work has applications in both biological tissue mechanics and biomimetics, being beneficial to both of them in its own way.

Several multiscale skeletal muscle models have been proposed to bridge fiber-level behavior with macroscopic tissue mechanics [23,35,36]. For example, Heidlauf et al. [14,37] developed a comprehensive chemo-electro-mechanical framework that couples excitation–contraction dynamics with continuum mechanics, while Lamsfuss et al. [29] presented a hierarchical constitutive formulation that accounts for the muscle microstructure within a continuum setting. Although these approaches provide valuable physiological insight, they either rely on phenomenological upscaling or incur significant computational cost due to the explicit resolution of electrophysiological and biochemical processes. One potential intermediary approach that has yet to be considered within the realm of composite-homogenization-based approaches is the combination of the phenomenological modeling of active forces, for example the three element Hill model, at the fiber level of a hierarchically based multiscale homogenization approach, thus enabling active forces to be transferred upwards through the micro-to-macro homogenization sequence without incurring the expensive chemo-electro-mechanical coupling computations.

The present work introduces a structured multiscale finite element framework based on explicit numerical homogenization at both the micro- and mesoscales. Inspired by multiscale modeling strategies widely used for textile and braided composite materials, periodic representative volume elements are employed to resolve fiber–matrix interactions and propagate their effective response systematically across hierarchical levels. This composite-inspired strategy enables a transparent and computationally efficient linkage between muscle microarchitecture and tissue-level mechanical behavior, without introducing additional constitutive complexity or biochemical coupling. As such, the proposed framework occupies a complementary niche between detailed chemo-electro-mechanical models and simplified phenomenological continuum descriptions.

It must be stressed that the current approach limits itself strictly to the mechanical properties of passive skeletal muscle subjected to tension. Such restrictions were made on purpose: the creation of a reliable theoretical background is an essential starting point prior to complicating the model by the inclusion of active processes, electromechanical coupling, or viscoelasticity. All three extensions are discussed in detail in the Section 4.

## 2. Materials and Methods

In order to correctly estimate and compute its behavior during the design phase and assess the muscles’ working stress and strain under active and inactive situations, numerical modeling was required. Muscle behavior is examined using multiscale modeling and analysis methods. Muscle conditions are recorded on micro, meso, and macro levels. Because muscle is made up of fiber bundles, multiscale modeling, and homogenization averaging are carried out for muscle bundles.

### 2.1. Modeling

To compute the effective elastic properties at the micro- and mesoscales, a series of independent strain-controlled loading cases were applied to the periodic representative volume elements. These included three normal strain modes (ε_11_, ε_22_, ε_33_) and three shear strain modes (ε_23_, ε_13_, ε_12_), applied individually while constraining all other strain components to zero. The resulting volume-averaged stress responses were used to populate the effective compliance matrix. The homogenization procedure is expressed using compact vector and matrix notation. The volume-averaged stress and strain vectors are defined as $\bar{\sigma }={\left[{\bar{\sigma }}_{11},{\bar{\sigma }}_{22},{\bar{\sigma }}_{33},{\bar{\sigma }}_{23},{\bar{\sigma }}_{13},{\bar{\sigma }}_{12}\right]}^{T},\bar{\varepsilon }={\left[{\bar{\varepsilon }}_{11},{\bar{\varepsilon }}_{22},{\bar{\varepsilon }}_{33},{\bar{\varepsilon }}_{23},{\bar{\varepsilon }}_{13},{\bar{\varepsilon }}_{12}\right]}^{T}$.

**Figure 1:**
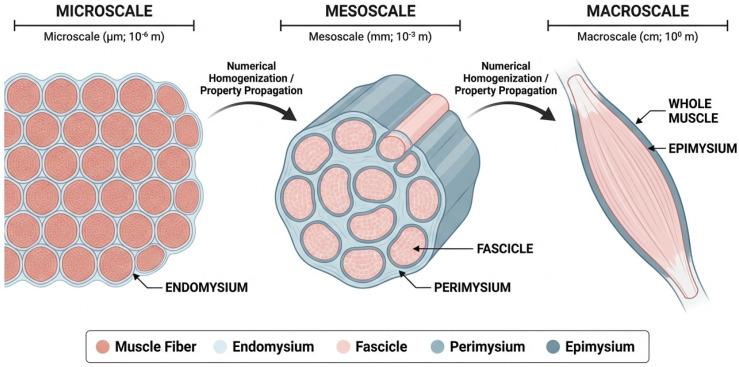
Muscle modeling.

These definitions are used consistently throughout the manuscript, and individual component expressions are omitted where possible for brevity. Modeling is a key step in covering the heterogeneous behavior of materials since it covers structure features [38]. Three scales were considered micro, meso, and macro (10^−6^, 10^−3^, 10^0^ m, respectively). The microscale model was based on fibers and endomysium mechanical properties to make fascicles and cover muscles’ micro-level details. In mesoscale, fascicles are modeled with perimysium (as a matrix). The mesoscale division is based on thin and thick regions of muscles in the arm body. Using those models repeatedly results in the entire macroscale muscle geometrical model, then a homogenized muscle model is prepared. Figure 1 illustrates the modeling approach at three different scales.

### 2.2. Microscale

In muscle modeling, fiber bundles form fascicles with Endomysium. The microscale model was set up based on fiber arrangement in the fascicle and fiber volume fraction (0.90). The fiber volume fraction at the microscale was set to V_f_ = 0.9, consistent with experimental and histological observations indicating that muscle fibers occupy the dominant fraction of the fascicle cross-section, with the endomysium forming a comparatively thin extracellular network [39]. Reported values of fiber volume fraction in healthy skeletal muscle typically range from approximately 0.85 to 0.95, depending on muscle type, age, and physiological condition. The chosen value therefore represents a physiologically realistic upper-bound estimate for normal adult muscle tissue.

**Figure 2:**
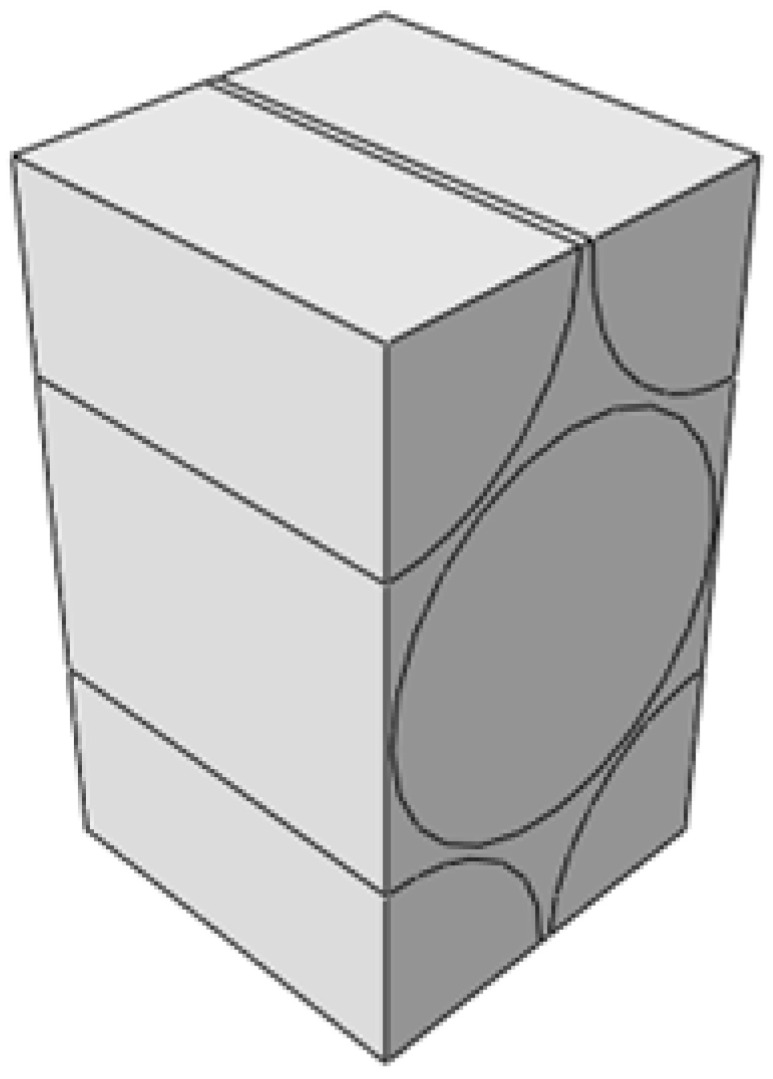
Microscale model for muscle analysis.

To assess the robustness of the homogenized response, preliminary sensitivity indicated that moderate variations in fiber volume fraction within this physiological range primarily affect the absolute stiffness level while preserving the overall nonlinear stress–strain trend. Consequently, V_f_ = 0.9 was adopted as a representative value to emphasize fiber–matrix load transfer mechanisms without loss of generality. The hexagonal distribution (as it covers fiber packing regions accurately) of fiber was considered in the fascicle to cover accurate details at the micro-level, as shown in Figure 2 [40,41]. The fiber bundle behavior is used to model fascicle bundles on the mesoscale. A hexagonal fiber packing arrangement was adopted at the microscale to represent the spatial organization of muscle fibers within a fascicle. From a geometrical standpoint, hexagonal packing provides the highest packing efficiency for cylindrical fibers, yielding a maximum theoretical packing density of approximately 0.907. This closely aligns with experimentally reported fiber area fractions in healthy skeletal muscle and allows the imposed fiber volume fraction to be achieved without artificial distortion of fiber geometry. Furthermore, histological studies have shown that muscle fibers tend to organize in locally regular patterns that are more accurately approximated by hexagonal rather than square arrangements. Accordingly, the hexagonal representative cell provides a quantitatively justified and computationally efficient idealization of fiber packing for numerical homogenization. According to previous studies, natural skeletal muscles possess random fascicle organization, variable diameters, branched structure, blood vessels, and non-uniform distribution of extracellular matrices. However, these physiological features are not considered in the current analysis because of the use of a periodic representative volume element. Hexagonal periodic arrangements have been used because they are simple to generate and analyze. Nevertheless, generating periodic RVE based on actual histology images would be one of the most important advancements for future studies.

### 2.3. Mesoscale

**Figure 3:**
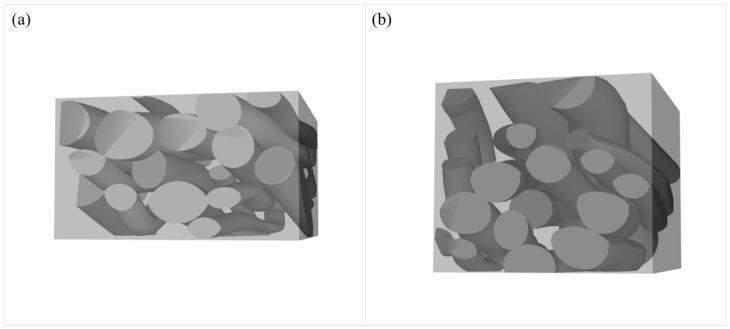
Mesoscale model of muscle, (**a**) Thin region, (**b**) Thick region.

The mesoscale model was prepared based on the fascicle distribution in muscle, and the curved fascicle was made to cover the contact region’s curve shape because it is a concerning and crucial part of the study. Mesoscale modeling is based on the position of muscles in the human arm body part (as it is considered a geometrical model for the current study). In the muscle position, fascicles are arranged in rows and columns (m×n), which generates a uniform pattern. To cover the whole region of muscle structure, thin and thick mesoscale models are prepared with fascicle and perimysium, as shown in Figure 3.

Shape, size, volume fraction, and fascicle count data were used to model the fascicles [42,43]. For the muscle model, one fascicle was created initially. By designating the fascicle’s midline with a number of separate control points, the direction of the fascicle in 3D space was created. Linear interpolation functions were used to connect these control points. To finish the mesoscale muscle model, additional fascicles were constructed at specific positions on the fascicle. Both scale models were created using Python scripts displayed in Figure 3 [44]. This approach to thin/thick classification of the regions is grounded in the generalized human arm muscle anatomy and is chosen as a demonstrative example of modeling. Personalized geometric representation of the muscle architecture based on medical images and including the details of fascicles’ curvature and bifurcations is the next step to take towards clinical implementation.

### 2.4. Macro Scale

The macro muscle model is modeled with epimysium as the arm dimension of muscle with the median proximal and distal region. This was done based on the anatomy and dimensions of the human arm. The length is considered according to the average young human hand length (30–35 cm) [45].

**Figure 4:**
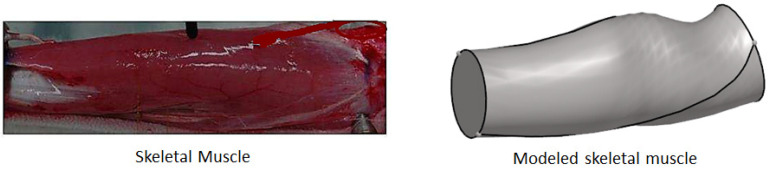
Skeletal muscle scan and current model.

Geometry was created using the muscle shown in Figure 4. Solid work (SW) modeling software generated a three-dimensional macroscale model. The mechanical properties of epimysium are taken from literature, and skeletal muscle models’ properties are taken from averaging mesoscale models.

All geometric and material assumptions were selected to balance physiological realism with computational tractability, consistent with the primary objective of establishing a transferable multiscale finite element workflow. Unless otherwise stated, tensor quantities are expressed in Voigt notation, and homogenized properties are reported with respect to the local fiber-aligned coordinate system.

### 2.5. Analysis

**Figure 5:**
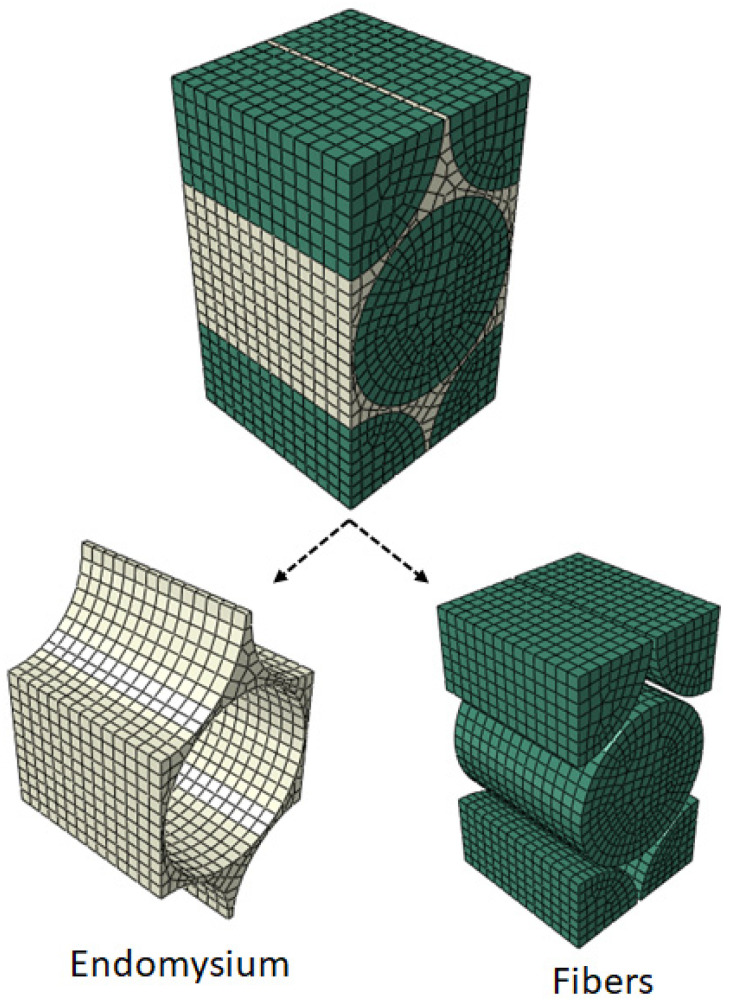
Meshed model for muscle analysis at microscale.

**Figure 6:**
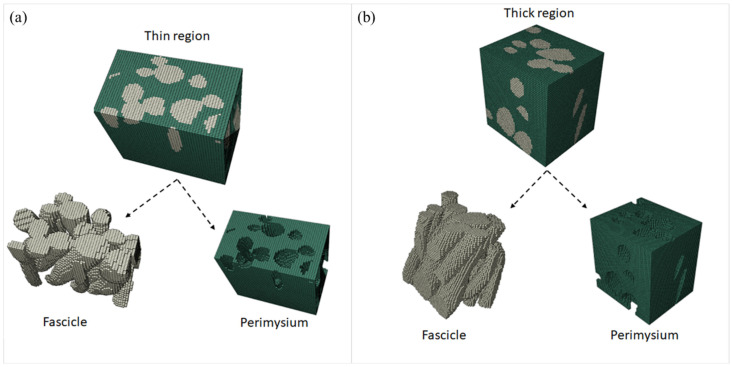
Meshed model at the mesoscale, (**a**) Thin region, (**b**) Thick region.

The micro and mesoscales have meshed with eight-node bilinear reduced integration C38DR solid elements. The number of elements in the meshed model is 7280 at the microscale, as shown in Figure 5. The number of elements for thin and thick regions’ mesoscale models are 21,815 and 22,484, respectively, as shown in Figure 6. The mechanical properties of constituents are shown in Table 1, where alpha, E, ν, d, and V_f/m_ are Ogden number, modulus, Poisson ratio, the diameter of the fiber, and volume fraction (fiber and matrix), respectively. The homogenized stress–strain behavior from the microscale is used for the fascicle in both the mesoscale model analyses. Hyperelastic behavior is considered when analyzing models.

All muscle constituents were modeled as nearly incompressible materials with Poisson’s ratio ν = 0.49, reflecting the high-water content and volume-preserving behavior of biological soft tissues under mechanical loading. This assumption is widely adopted in computational muscle mechanics and soft tissue modeling and is particularly appropriate for passive deformation regimes dominated by extracellular matrix response. To ensure numerical stability under near-incompressible conditions, reduced-integration hexahedral elements were employed at all scales. This choice mitigates volumetric locking while maintaining computational efficiency. Although mixed u–p formulations offer an alternative strategy for enforcing incompressibility, the present formulation was found to be stable and accurate for the strain ranges considered, as evidenced by smooth stress–strain responses and close agreement with literature benchmarks.

The constitutive modeling decisions made for this work require a clear rationale. The use of a hyperelastic, nearly incompressible constitutive model is proven for passive muscle biomechanics. This framework is also suitable for the passive tensile deformation range, as in this case, the main nonlinearity stems from the interaction of fibers with matrix but not from any activation processes or time-dependent properties. Fiber recruitment, anisotropic activation, sarcomere dynamics, and strain-rate effects belong to active or viscous properties that do not apply to the present passive elastic material framework. Concerning the Poisson ratio of ν = 0.49: This number accounts for the near incompressibility of hydrated biological soft tissues in their passive state. Although individual Poisson ratios could have been assigned to different constituents of muscle, at present, there are no well-supported distinct Poisson ratios for endomysium, perimysium, and epimysium. It can be regarded as a future goal.

The boundary and load conditions are implemented in the Abaqus finite element code. Periodic boundary conditions are enforced on all faces of the representative volume elements (RVEs) at the micro- and mesoscales. The use of periodic boundary conditions is theoretically sound for homogenization of periodic materials since it guarantees traction continuity and displacement compatibility along unit cell boundaries and thus provides an exact homogenization process devoid of any artificial boundary condition issues. After imposing each of the six independent unit strains, the effective compliance tensor is constructed by performing the homogenization. At the macroscale level, the material is subjected to tensile loading at one end of the muscle model, with the other end fixed, as in the experiments used for validation purposes. It is understood that in vivo loading conditions are far more complicated, including tendon mechanics, heterogeneous activation, inter-muscle interaction, and even the kinematics from adjacent tissues. Such boundary conditions are valid for the present application in order to develop the homogenization framework. In future work, tendon attachments can be modeled and coupled with musculoskeletal models such as OpenSim, as well as heterogeneous boundary conditions from imaging or motion-capture data. In order to check the convergence and thereby the independence of the simulated stress–strain behavior on the mesh size, an extensive investigation was carried out at both the micro- and mesoscales through a set of tests with three types of meshes, namely, (i) coarse mesh having around half the number of elements than the baseline case, (ii) the baseline mesh as shown in Figure 5 and Figure 6, and (iii) a fine mesh which had around double the number of elements than the baseline case. For the uniaxial tensile test, the volume averaged stress at the representative strain level was examined for the three refinement levels. The difference between the baseline and fine mesh results were within 1.5% and 2% at the micro- and mesoscales, respectively, ensuring mesh-independent results in the current range of strains. Finally, at the mmacroscale the model with 29,512 elements exhibited smooth and continuously increasing stress–strain curves without showing volumetric locking or hour glassing effects, in agreement with the stabilization of the reduced integration used in C3D8R elements.

After the analysis, the calculations proceeded to calculate the material properties of the region. The constitutive global strain–stress correlation is shown below in Equation (1) for micro- and mesoscale regions:

(1) $$\bar{{ɛ}_{i}}=S_{ij}{\bar{\sigma }}_{j}$$

where S_ij_ is the effective compliance matrix, and ${\bar{\sigma }}_{j}$ and $\bar{{ɛ}_{i}}$ are the global averaging stress and strain, respectively, which are defined by Equations (2) and (3).

(2) $${\bar{\sigma }}_{ij}=\frac{1}{V}\int \sigma_{ij}dV=\;\frac{\sum_{n=1}^{n_{e}}\left(\sigma_{ij}^{e}V_{n}^{e}\right)}{\sum_{n=1}^{n_{e}}V_{n}^{e}}$$

(3) $${\bar{\varepsilon }}_{ij}=\frac{1}{V}\int \varepsilon_{ij}dV=\frac{\sum_{n=1}^{n_{e}}\left(\varepsilon_{ij}^{e}V_{n}^{e}\right)}{\sum_{n=1}^{n_{e}}V_{n}^{e}}\;(i,\;j=1,2,3\ldots )$$

where $\sigma_{ij}^{e}$ and $\varepsilon_{ij}^{e}$ denote the stress and strain of elements. $n_{e},$ $V_{n}^{e}$, and V are total number of elements, elements’ volume, and regions’ volume, respectively. In Equation (3), $\varepsilon_{ij}$ is a known variable in the FEM to apply to the PBC. Based on the definition, the effective elastic properties can be obtained by the integral repetitive-region model, i.e., $\left[\bar{\sigma }\right]={\left[{\bar{\sigma }}_{ii}\left(i=1,2\;and\;3\right),{\bar{\sigma }}_{jk}(jk=23,13,12)\right]}^{T},\left[\bar{\varepsilon }\right]={\left[{\bar{\varepsilon }}_{ii}\left(i=1,2\;and\;3\right),{\bar{\varepsilon }}_{jk}(jk=23,13,12)\right]}^{T}$. Further calculations of elastic constants are based on Equations (4).

(4) $$E_{i}=\frac{{\bar{\sigma }}_{i}}{{\bar{\varepsilon }}_{i}},\;G_{ij}=\frac{{\bar{\sigma }}_{ij}}{{\bar{\varepsilon }}_{ij}},\;\mu_{ij}=-\frac{{\bar{\varepsilon }}_{i}}{{\bar{\varepsilon }}_{j}},\;(i,\;j=1,2,3\ldots )$$

(5) $$S_{ij}=\bar{{ɛ}_{i}}{{\bar{\sigma }}_{j}}^{-1}$$

(6) $$\begin{array}{c} S_{ij}=\left(\begin{array}{cccccc} S_{11} & S_{12} & S_{13} & S_{14} & S_{15} & S_{16}\\ S_{21} & S_{22} & S_{23} & S_{24} & S_{25} & S_{26}\\ S_{31} & S_{32} & S_{33} & S_{34} & S_{35} & S_{36}\\ S_{41} & S_{42} & S_{43} & S_{44} & S_{45} & S_{46}\\ S_{51} & S_{52} & S_{53} & S_{54} & S_{55} & S_{56}\\ S_{61} & S_{62} & S_{63} & S_{64} & S_{65} & S_{66} \end{array}\right)\\ =\left(\begin{array}{cccccc} 1/E_{11} & -v_{21}/E_{22} & -v_{31}/E_{33} & 0 & 0 & 0\\ -v_{12}/E_{11} & 1/E_{22} & -v_{32}/E_{33} & 0 & 0 & 0\\ -v_{13}/E_{11} & -v_{23}/E_{22} & 1/E_{33} & 0 & 0 & 0\\ 0 & 0 & 0 & 1/G_{23} & S_{45} & 0\\ 0 & 0 & 0 & 0 & 1/G_{13} & 0\\ 0 & 0 & 0 & 0 & 0 & 1/G_{12} \end{array}\right) \end{array}$$

(7) $$\left[C_{ij}\right]={\left[S\right]}_{ij}^{-1}$$

Thus, regions’ stiffness matrices are obtained. Then, the stiffness matrix from each region is aggregated into the final combined mesoscale model. The summation of two regions was given final mechanical properties using the volume averaging method. The final calculated mechanical properties will be obtained by Equation (8).

(8) $$\begin{array}{c} C_{final}=\sum_{1}^{n}V_{n}{\bar{C}}_{ij(n)}\\ S_{final}={C_{final}}^{-1} \end{array}$$

(V_n_ = volume fractions of thick and thin regions of the macrostructure of muscle)

**Figure 7:**
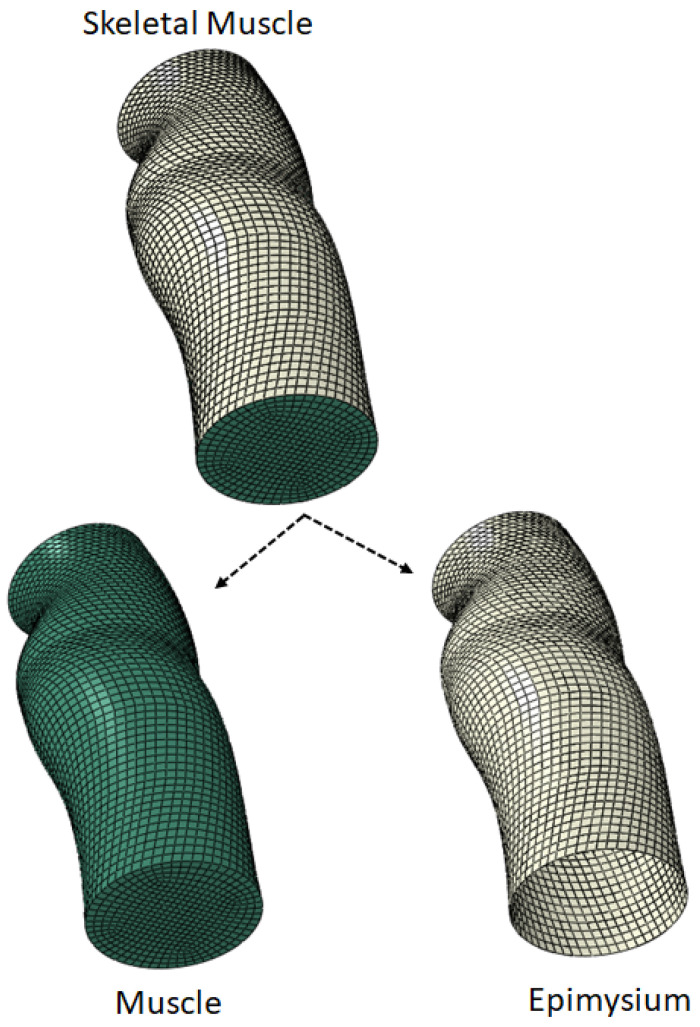
Macroscale meshed model of muscle.

The calculated homogenized mechanical properties of skeletal muscles were used for selected arm skeletal muscle. FEM has been performed on muscle with the homogenized response of muscle and epimysium properties. The FE analysis model is shown in Figure 7. That has meshed with 20 nodes of hexahedral C3D8R elements. The total number of elements in the model is 29,512, and a fine mesh is applied to the ECM and muscle region. Mesh size and alignment are affirmed to capture heterogeneity. The boundary and load terms were employed for the analysis. A tension load is applied with a fixed support on macroscale geometry to check its stretch response.

## 3. Results and Discussion

Muscle structures incorporate complexity in some way. With considerably simpler approximations of the actual muscle, it would not be able to obtain the same or even better outcomes. The literature has numerous instances when very simple concepts have led to incredibly complicated incidents. Using 1D-line components for muscles with simplistic contraction models, several gait analysis issues can be resolved. This basic model has considered 1D or 2D structures to capture activity at a shallow level. Getting actual 3D to more inside micro details led to multi-level 3D FEM in which different length scales are integrated [48,49,50]. It has been demonstrated that this process can be used to calculate macroscopic structure behavior by looking at the micro/mesoscale. If it is needed to understand how muscle adapts to variations in the mechanical state, it requires knowing local stresses and strains. Previous finite-element formulations, such as a discrete model [51] or a different method of continuum mechanics [52], have the drawback of fulfilling the volume-conserving situation or enlarging the model to 3D and high computation time due to asymmetricity. After understanding the previous work, the strategy needs to develop to analyze muscle behavior with an efficient modeling and analysis approach. This work examines the mechanical characteristics of the skeletal muscle at various levels of hierarchy according to the region by employing a multiscale, multicell strategy.

**Figure 8:**
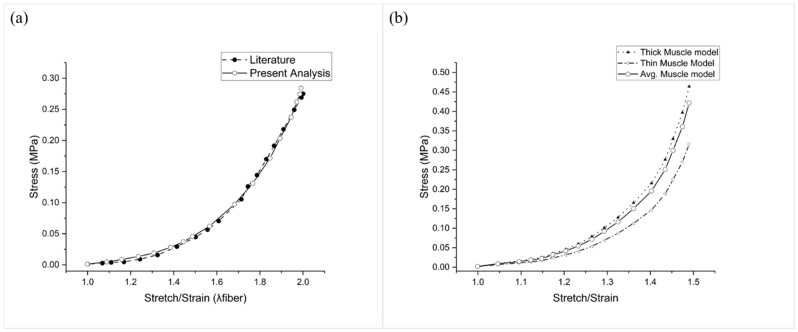
(**a**) Stress–strain curve of microscale response, and (**b**) stress–strain curve of muscles’ region and average response.

Fibers, endomysium to fascicle, fascicle, and perimysium to muscle behavior are covered, and then at the last level, muscle and epimysium structure are analyzed in the tension loading and activation scenario. This research aims to demonstrate the capabilities of the modeling and analysis approach by using FEA to evaluate the action of skeletal muscles on a biological structure model. It is assumed that the degree of activity is constant along the fiber for fully active muscle fibers. Models (at the micro, meso, and macro levels) are created along the fiber direction to determine stress/strain development in the structure. Understanding displacement–force (strain–stress) behavior under tensile loading is crucial to characterize muscle behavior. The microscale analysis shows homogenized behavior for mesoscale fascicles; then mesoscale homogenized properties are obtained for the thick (proximal) and thin (distal) regions. The mesoscale results are combined by the volume averaging method. Then, the final homogenized responses are obtained and used for skeletal muscles’ macroscale analysis. The stress–strain responses are verified at the microscale by comparing the literature, as shown in Figure 8a; the difference in percentage from the literature is approx. 1.4 [29].

**Figure 9:**
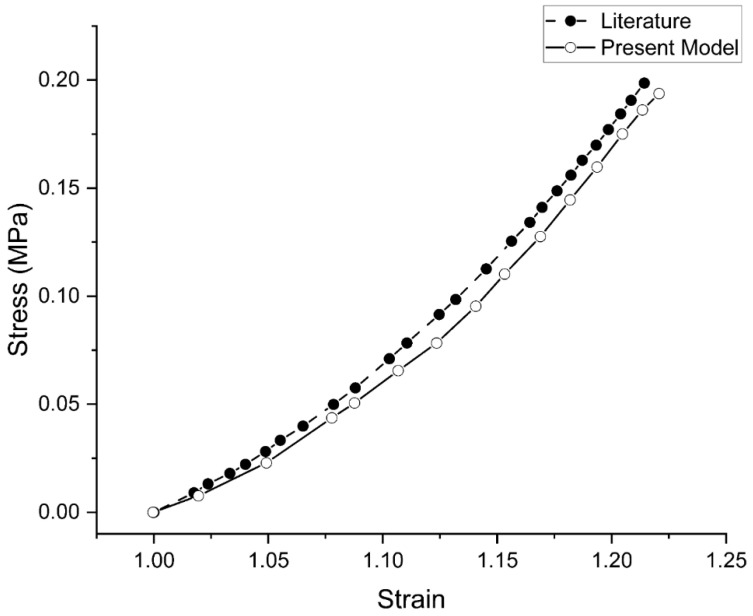
Macroscale results graph.

It is critical to differentiate benchmark verification from independent experimental validation of the model. The model predictions in Figure 8a and Figure 9 represent benchmark verification, whereby the predicted stress–strain response is compared against previous numerically and experimentally determined values in the literature to verify the internal validity of the proposed homogenization procedure. Independent experimental validation, which includes performing experiments on subject-specific specimens in controlled conditions, is not covered in this study but is highlighted as one of the most important areas for future studies. To account for model uncertainties to some extent, a preliminary sensitivity analysis was performed by varying the fiber volume fraction (V_f_) in the physiological range of values (0.85 to 0.95) and varying the Ogden constant (α) by ±15%. It is observed that the stress–strain response remains consistent with variations in the peak stress values being approximately 8–12% in either direction.

After verifying the microscale model behavior and its homogenized response, it is used for mesoscale analysis. The unit cell’s average stresses, which are brought on by the fiber and endomysium strains, are known as fascicle stresses. The starting length of every sarcomere is considered to correspond to the resting length. In endomysium, stress develops during tension, increasing gradually for minor tensile strains and dramatically for strains greater than 50%. A load transfer transition within the fascicle is readily discernible at 30% tensile strain. Endomysium contributes significantly to strain resistance for high strains, according to the substantially nonlinear behavior of endomysium and the stress–strain curve for higher strains. The stress–strain graph in Figure 8b shows the stress–strain variation in the thin and thick regions with an average calculated behavior that is 0.35 MPa. The endomysium carries most of the load, which causes stress concentrations almost ten times greater than the average stress in the fascicle. A post-processing evaluation of the microscale unit cell indicates that, under tensile strains exceedingly approximately 30%, the endomysium sustains a disproportionately large share of the load relative to its volume fraction. Specifically, while occupying roughly 10% of the microscale volume, the endomysium contributes on the order of 40–60% of the total averaged stress within the representative cell at higher strain levels. This amplification reflects the nonlinear stiffening behavior of the extracellular matrix and the progressive transfer of load from muscle fibers to the surrounding connective tissue network. The total stress is not zero at fiber lengths where they can no longer be engaged because of the passive forces in fibers during stretching. The force created within a fascicle, which is the force within a unit cell, provides information about how muscle fibers and connective tissue interact. The tensile force in the endomysium already significantly contributes to the fascicle tension at this stretch level.

**Figure 10:**
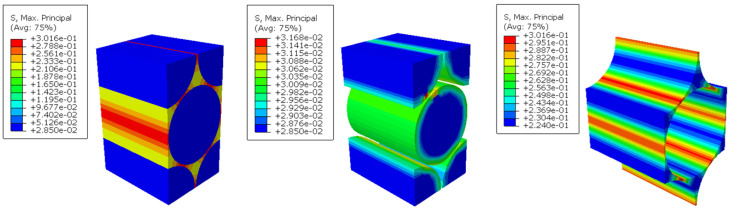
Microscale behavior.

**Figure 11:**
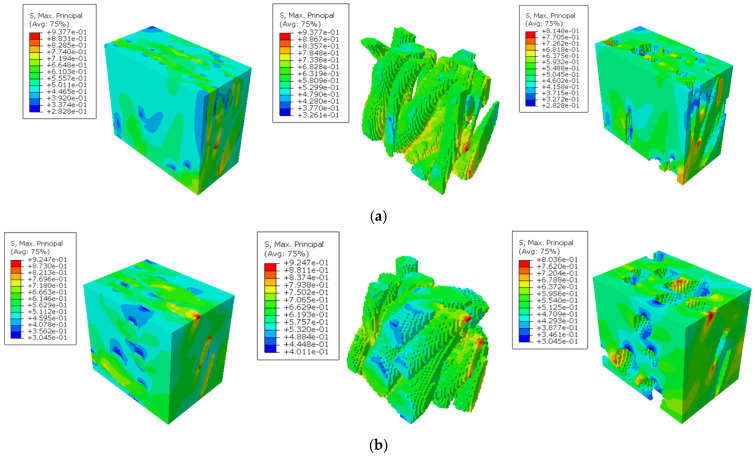
Mesoscale behavior, (**a**) Thin region, (**b**) Thick region.

The microscale nephogram results are presented in Figure 10. The results are shown for the combined model (left), followed by the muscle fiber and endomysium (right). Figure 11a,b shows the mesoscale analysis contour variation results for thin and thick reasons. The results are shown for the combined model (left), then the fascicle and matrix (right). It can be seen that the thin region has a slightly higher stiffness than the thick region due to the resisting cross-section area/volume. The obtained response is useful for macroscale analysis.

**Figure 12:**
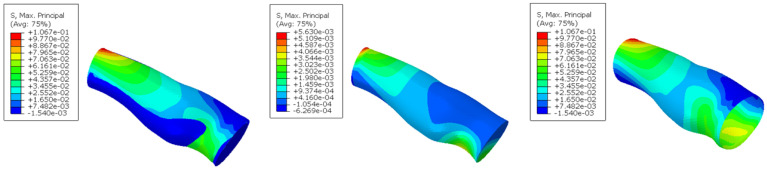
Macroscale mechanical response of the skeletal muscle.

Macroscale stress contour variation results are presented in Figure 12. The stress variation can be seen in distal and proximal regions. The results show combined, homogenized, and epimysium from left to right. The stress is higher at the edge of the muscle at the epimysium zone; the fascicle resists and opposes the stretch response. Those are compared with the literature, as shown in Figure 9, and the approximate percentage difference is 3 percent [53]. The nonlinear stress–strain graph supports the material’s nonlinearity as the input material model and material data are taken from the experimental literature to twin and replicate the virtue of the analysis strategy, and the design 3D model is taken as a scan condition to make a realistic design method for analysis. The present validation focuses on passive tensile behavior; extension to multiaxial loading, time-dependent effects, and active contraction will be addressed in future work. In particular, the validation of such models would focus on multiaxial loading regimes, such as transverse compression and simple shear, based on experimental results from mechanical testing of fresh or fixed muscle specimens and in vivo measurement of deformations from imaging-based elastography and diffusion tensor MRI.

Table 2 summarizes key differences between representative multiscale skeletal muscle models and the present framework, highlighting distinctions in scale resolution, homogenization strategy, and computational complexity.

From a clinical perspective, the multiscale nature of the framework enables a mechanistic interpretation of how microstructural alterations observed in disease or aging translate into changes in macroscopic muscle stiffness and extensibility [55,56]. For example, increased connective tissue content or stiffening at the endomysial or perimysial levels—commonly reported in fibrotic and dystrophic muscles—can be introduced directly into the micro- or mesoscale models, allowing their isolated and combined effects on whole-muscle mechanics to be quantified.

Such an approach moves beyond descriptive correlations between pathology and muscle stiffness by providing a physics-based explanation of how specific structural changes contribute to functional impairment. As seen in the sensitivity analysis performed in section three, endomysial stiffness and the fiber Ogden nonlinearity constant prove to be the most important parameters in the behavior of muscles from a mechanical perspective; this information is relevant in considering the effects of fibrosis where an increase in ECM stiffness leads to increased passive resistance and in examining sarcopenia due to a decrease in fiber volume fraction.

### 3.1. Rehabilitation Modeling

For rehabilitation modeling, the suggested model offers a mechanism-based foundation, which is not yet a clinical tool but can be developed into one with proper extrapolation and experimentation to assist in creating and analyzing treatment approaches designed to affect passive muscle stiffness, including stretches and physical therapy treatments after surgery. For this approach, personalized geometry and tissue properties, along with the associated loading, are also needed. By adjusting connective tissue properties at different hierarchical levels, the model can be used to predict how targeted interventions may influence regional muscle compliance and load redistribution, thereby informing therapy intensity and duration.

### 3.2. Surgical Planning

For surgical planning, particularly in procedures involving muscle lengthening, tendon transfer, or compartment release, understanding how stress redistributes within muscle tissue is critical. The point must be made that the above-presented modeling approach is not yet considered to be a surgical planning tool because no specific data from the patients, such as geometry of the organs, boundary conditions, etc., were used in simulations. Below there will be discussed a mechanical reasoning explaining why this approach could be used for surgical planning. The present multiscale approach offers a means to assess how changes at the tissue boundary (e.g., epimysium or surgical incision sites) propagate internally to affect fascicle and fiber-level stresses, potentially aiding in the evaluation of post-operative stiffness and injury risk.

### 3.3. Muscle Disease Progression (Fibrosis and Dystrophy)

This model structure is suitable—but has not yet been shown to be computable—for investigating the progression of muscle diseases like fibrosis and muscular dystrophy. No pathological tissue measurements, no abnormal ECM stiffness data, and no imaging data of diseased tissue have been incorporated into the current simulations, and what follows is a hypothesis based on the model architecture. Progressive increases in connective tissue stiffness or volume fraction can be introduced incrementally at the micro- and mesoscales, enabling simulation of disease evolution and assessment of its mechanical consequences at the organ level. This capability provides a foundation for hypothesis-driven investigations into how early microstructural changes may serve as precursors to clinically observable dysfunction.

By explicitly linking muscle microstructure to macroscopic mechanical behavior, the proposed multiscale framework provides a mechanistic bridge between experimental observation, computational modeling, and clinical interpretation of skeletal muscle function.

The pronounced load-bearing role of the endomysium highlighted by the present results has direct implications for pathological conditions characterized by connective tissue remodeling, such as muscle fibrosis. In fibrotic muscle, increased collagen content and cross-linking are known to elevate endomysial stiffness, which would, within the present framework, shift an even greater proportion of load to the extracellular matrix at lower strain levels. This mechanism provides a structural explanation for the elevated passive stiffness and reduced extensibility observed clinically in fibrotic and dystrophic muscles. Consequently, the proposed multiscale framework offers a mechanistic platform for investigating how microstructural alterations in connective tissue propagate to whole-muscle mechanical dysfunction, beyond what can be captured using homogenized single-scale constitutive models.

## 4. Conclusions

This study presents a multiscale finite element modeling framework for analyzing the mechanical behavior of skeletal muscles by linking microstructural organization and macroscopic responses through numerical homogenization. The approach integrates three hierarchical levels, muscle fibers and endomysium at the microscale, fascicles and perimysium at the mesoscale, and the whole muscle with epimysium at the macroscale, all within a consistent computational framework. By resolving fiber–matrix interactions at lower scales and propagating their effective behavior upward, the framework establishes a connection between the muscle microstructure and tissue-level mechanics. Finite element simulations at each scale demonstrated nonlinear elastic stress–strain behavior under tensile loading, and the homogenized responses showed good agreement with the literature results, with deviations on the order of a few percent. These findings indicate that the proposed homogenization strategy effectively captures the key features of skeletal muscle mechanics while maintaining the computational efficiency. The principal contribution of this study lies in demonstrating a structured and extensible multiscale finite element methodology that can be implemented using standard analytical tools. The framework is flexible and can be adapted to different muscle geometries, architectural variations, or changes in connective tissue properties, making it suitable for parametric and comparative analyses. The future research will take the present framework forward through several well-defined paths. First, active elements (modeled based on either Huxley-type sliding filament model or phenomenological models for activation) will be incorporated into the fiber level, so that active stress generation and electromechanical interaction can be modeled. Second, viscoelastic models will be used to account for the rate dependency as well as the time dependency of the behavior (such as stress relaxation or creep due to sustained loading). Third, subject-specific muscle geometries obtained using either MRI or CT scan will be incorporated to replace the idealized geometry of the arm. All these enhancements will increase the utility of the proposed modeling framework in studying the changes associated with physiology, injury, rehabilitation, and disease conditions in muscle mechanics.

## Figures and Tables

**Table 1:** Muscle constituents’ mechanical properties [6,29,46,47].

Material Constituents	Mechanical Properties
Matrix for microscale (Endomysium)	E = 0.24 MPa, ν = 0.49, V_m_ = 0.1
Fiber (Muscle Fiber)	E = 0.047 MPa, ν = 0.49, V_f_ = 0.9, α = 7.95, d_fiber_ = 0.047 mm
Matrix for mesoscale (Perimysium)	E = 1.5 MPa, ν = 0.49, V_m_ = 0.15
Matrix for macroscale (Epimysium)	E = 2 MPa, ν = 0.49, V_m_ = 0.08

**Table 2:** Comparison of representative multiscale skeletal muscle modeling approaches.

Scales Resolved	Upscaling Strategy	Constitutive Description	Computational Cost	Validation	References
Sarcomere → Fiber → Muscle	Direct multiscale coupling	Chemo-electro-mechanical, active contraction	High	Force–length & contraction data	[14]
Fiber → Fascicle → Muscle	Hierarchical constitutive modeling	Hyperelastic, anisotropic	Moderate–High	Experimental stress–strain	[29]
Fiber → Fascicle	Micromechanical FE	Passive elastic	Moderate	Shear & tensile response	[22,54]
Fiber → Fascicle → Whole muscle	Explicit periodic FE homogenization (micro & meso)	Hyperelastic, composite-inspired	Moderate/Low	Literature benchmarks (1–3%)	Present study

## Data Availability

Data is available upon reasonable request to the corresponding author.
